# Diverse mantle components with invariant oxygen isotopes in the 2021 Fagradalsfjall eruption, Iceland

**DOI:** 10.1038/s41467-022-31348-7

**Published:** 2022-06-29

**Authors:** I. N. Bindeman, F. M. Deegan, V. R. Troll, T. Thordarson, Á. Höskuldsson, W. M. Moreland, E. U. Zorn, A. V. Shevchenko, T. R. Walter

**Affiliations:** 1 Department of Earth Sciences, University of Oregon, Eugene, OR USA; 2 Department of Earth Sciences, Section for Natural Resources & Sustainable Development, Uppsala University, Uppsala, Sweden; 3 Centre of Natural Hazards and Disaster Science (CNDS), Uppsala University, Uppsala, Sweden; 4 Faculty of Earth Sciences, University of Iceland, Reykjavík, Iceland; 5 Institute of Earth Sciences, University of Iceland, Reykjavík, Iceland; 6 GFZ German Research Centre for Geosciences, Potsdam, Germany

**Keywords:** Geochemistry, Volcanology, Natural hazards

## Abstract

The basalts of the 2021 Fagradalsfjall eruption were the first erupted on the Reykjanes Peninsula in 781 years and offer a unique opportunity to determine the composition of the mantle underlying Iceland, in particular its oxygen isotope composition (δ^18^O values). The basalts show compositional variations in Zr/Y, Nb/Zr and Nb/Y values that span roughly half of the previously described range for Icelandic basaltic magmas and signal involvement of Icelandic plume (OIB) and Enriched Mid-Ocean Ridge Basalt (EMORB) in magma genesis. Here we show that Fagradalsfjall δ^18^O values are invariable (mean δ^18^O = 5.4 ± 0.3‰ 2 SD, *N* = 47) and indistinguishable from “normal” upper mantle, in contrast to significantly lower δ^18^O values reported for erupted materials elsewhere in Iceland (e.g., the 2014–2015 eruption at Holuhraun, Central Iceland). Thus, despite differing trace element characteristics, the melts that supplied the Fagradalsfjall eruption show no evidence for ^18^O-depleted mantle or interaction with low-δ^18^O crust and may therefore represent a useful mantle reference value in this part of the Icelandic plume system.

## Abstract (web summary)

The 2021 eruption in the Reykjanes Peninsula of Iceland was the first in 800 years and was supplied by melts from diverse mantle source domains with near-identical oxygen isotope ratios, providing a unique insight into the Icelandic mantle plume.

## Introduction

**Fig. 1:**
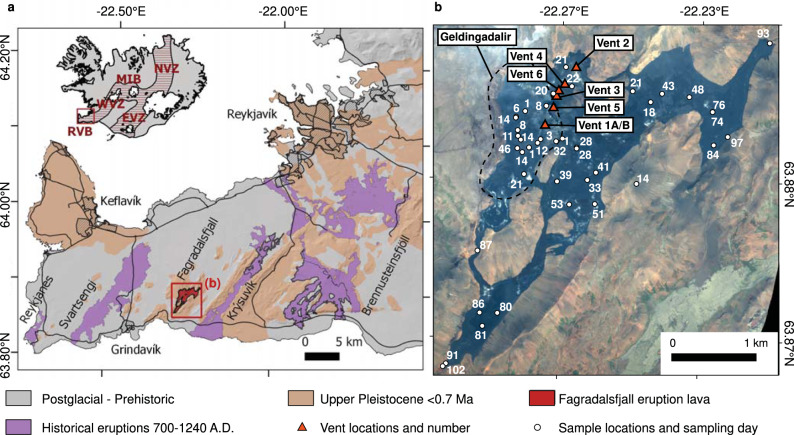
The Fagradalsfjall eruption site on the Reykjanes Peninsula. **a** Inset shows the main rift zones on Iceland (EVZ = Eastern Volcanic Zone; MIB = Mid-Iceland Belt; NVZ = Northern Volcanic Zone; RVB = Reykjanes Volcanic Belt; WVZ = Western Volcanic Zone). Map of Reykjanes Peninsula shows historical (purple; late Holocene) and prehistoric (brown and grey; early Holocene, Postglacial and Pleistocene) eruptions as well as the extent of the lava field of the 2021 Fagradalsfjall eruption (red). The base map is based on data from the National Land Survey of Iceland (www.lmi.is) with geological data after ref. ^48^. **b** Close-up satellite map of the red box in panel (a) showing sampling locations numbered by the days since the start of the eruption (white dots). The positions of the eruptive vents are also marked (red triangles) and numbered in order of appearance. Background image is an orthophoto generated with the Plèiades tri-stereo satellite (www.earth.esa.int/eogateway/missions/pleiades).

Iceland has long been viewed as the product of a mantle plume intersecting the mid-Atlantic ridge on the Eurasian and North American plate boundary. The Reykjanes Peninsula (RP) in Western Iceland is not only a volcanically active plate boundary functioning as the onshore extension of the Reykjanes Ridge, but it also hosts about 70% of Iceland’s population, including the Greater Reykjavík area, the Svartsengi and Reykjanes geothermal power plants, and Keflavík international airport. The RP is a transform zone where highly oblique spreading at a rate of 18.8 mm/year gradually pushes the North American and Eurasian plates apart^1^. Until March 19th 2021, when a low-intensity effusive basaltic eruption began at Fagradalsfjall (Fig. 1), the peninsula had been volcanically dormant for 781 years^2^. Assessment of eruption history on the peninsula over the last 4000 years indicates that the RP experienced three distinct eruption periods separated by ca. 800 year-long repose periods. Each period lasted for 200–400 years, featuring periodic eruptions that were confined to narrow SSW to NNE trending lineaments and fracture systems aligned in an *en echelon* fashion across the peninsula (Fig. 1a). In the last eruption period, which began around 800 CE and lasted until 1240 CE, the earliest eruptions took place at the east end of the peninsula and a westward migration of subsequent eruption sites followed (see ref. ^2^; Fig. S1). However, whether this is a pattern within individual eruption periods remains uncertain.

The RP has also been the location of large shallow tectonic earthquakes with magnitudes up to M6 that are typically confined to the upper crust to a depth of ca. 6 km^3,4^. In total, 39,500 earthquakes, as well as a period of strong inflation, were detected in 2020 in a region just 8 km to the west of the Fagradalsfjall eruption site^5^. This seismogenic zone is underlain by a brittle-ductile transition at 6–7 km depth^6^ and the Moho at around 15 to 20 km depth^4,5^. In light of the volcanic history and the geodynamic setting of the RP, the 2021 Fagradalsfjall eruption most likely represents an onset of a new eruption period, which may last centuries. Surface deformation and crustal seismicity on the RP can be explained by geothermal fluid mobilisation and ascent into shallow sealed zones along with instability of a magma reservoir in the lower crust or upper mantle between 15 and 20 km depth^5^. Therefore, it is important to understand the processes and composition of the mantle sources that fed the Fagradalsfjall eruption (and by implication also other parts of the RP), to better assess magma and hydrothermal fluid production in this area from a geological, volcanological and economic point of view.

Despite decades of research, there is a long-standing debate concerning the Icelandic geodynamic setting and the exact nature of the plume components involved in magma genesis. Geochemically enriched as well as depleted components (defined based on trace elemental ratios such as Zr/Y, Nb/Zr, Nb/Y as well as radiogenic isotopic ratios) have been suggested to be delivered to the sub-Icelandic magmatic system by a deep plume source under Iceland (e.g., refs. ^7–10^). Stable isotopes of oxygen (^18^O/^16^O ratios expressed in standard delta notation as δ^18^O values in ‰) are a particularly robust tracer of crustal and mantle inputs to Icelandic magmatism because (i) oxygen is a major (ca. 50 wt%) component of magmatic rocks, (ii) oxygen isotope ratios are only minimally fractionated at the high temperatures characteristic of mantle processes, but undergo large fractionations at low-temperature surface conditions, and (iii) mantle and crustal components have distinctly different oxygen isotope ratios (e.g., refs. ^11,12^), making it possible to effectively trace crustal inputs to mantle-derived melts. Oxygen isotope studies on Icelandic rocks and minerals have shown that many Icelandic basalts, including their crystals and melt inclusions, display a large degree of oxygen isotope diversity spanning several permille from around +2 to +6‰ (e.g., refs. ^13–21^). This range is far beyond the variation that is commonly ascribed to magmatic processes and significantly different to “normal” ambient upper mantle, which has relatively uniform δ^18^O values of ca. 5.5‰ (i.e., Mid-Ocean Ridge Basalt, MORB; ref. ^22^). The reasons for this large degree of oxygen isotope diversity in Iceland remain uncertain and the question of whether the observed diversity is the result of an original mantle source with heterogeneous δ^18^O values or crustal assimilation involving hydrothermally altered, low-δ^18^O basaltic crust, or a combination of the two, remains unresolved (cf. ref. ^23^).

This paper contributes to the Icelandic oxygen isotope debate via geochemical characterisation of pristine and relatively primitive eruption products from the 2021 Fagradalsfjall eruption, where we have identified involvement of at least three different mantle source components in magma genesis. We present major and trace element data as well as δ^18^O values for a suite of Fagradalsfjall samples and test for significant diversity in δ^18^O values among them. Our samples cover a time-span of 160 days from the start of the eruption in March until August 2021, thus allowing us to establish the initial magmatic composition and to test also for temporal changes in δ^18^O values as the eruption progressed. Moreover, we analysed a subset of our samples for Δ′^17^O (this parameter reflects linearised deviations in ‰ of ^17^O/^16^O ratios relative to a mass-dependent ^17^O/^16^O versus ^18^O/^16^O fractionation line with reference slope of 0.5305; see Methods), stable isotopes of hydrogen (^2^H/^1^H ratios expressed in standard delta notation as δD values in ‰), and water contents (H_2_O in wt%) to further constrain Fagradalsfjall magma source characteristics. In brief, the Fagradalsfjall samples analysed here display variations in their incompatible trace element ratios that can be linked to distinct mantle components. As a consequence of these trace element variations, we now have the unique opportunity to test for potential coupling of δ^18^O values to various mantle components and to assess hypotheses relating to the oxygen isotope composition of the mantle underlying the Reykjanes Peninsula.

## Results

### The 2021 Fagradalsfjall eruption

**Fig. 2:**
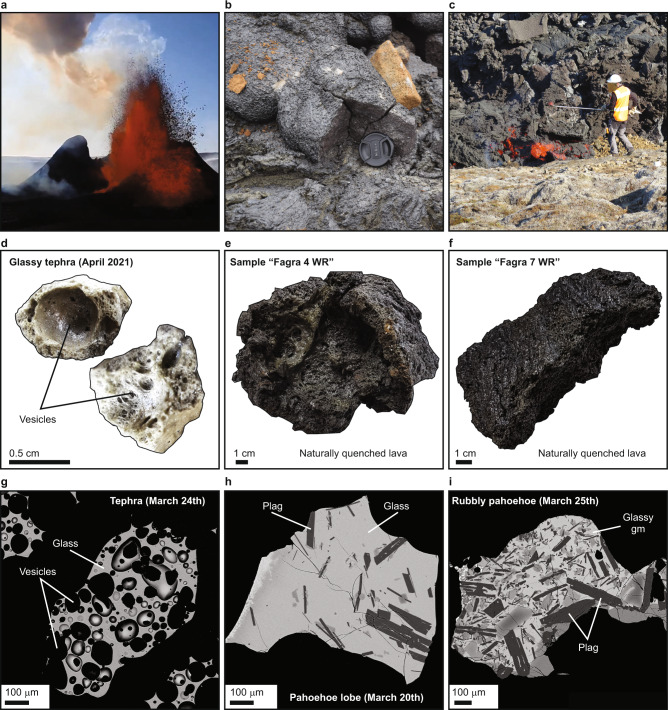
Fagradalsfjall 2021 eruption and samples. **a**–**c** Photographs of various phases of activity during the Fagradalsfjall eruption in April and June 2021. **a** Typical lava fountaining during an eruption episode in May 2021. **b** Field appearance of a pahoehoe lobe. **c** Sampling at a rubbly pahoehoe lava front. **d**–**f** Hand sample photographs showing the typical appearance of tephra and quenched lavas employed in this study. **g**, **h** Back-Scatter Electron (BSE) images of early erupted products ranging from glassy, highly vesiculated tephra (**g**) to more extensively crystallised material characteristic of rubbly pahoehoe lavas (**i**). Abbreviations: Gm, groundmass; Plag, plagioclase.

Although the Fagradalsfjall lineament features both Weichselian subglacial volcanic edifices and Holocene lava formation, prior to 2021 it had not erupted for over 2400 years. Therefore, previous activity predates the settlement of Iceland in the nineth century CE (e.g., ref. ^24^). On March 19th 2021 a low-intensity effusive basaltic eruption, the first on the Reykjanes Peninsula for 781 years (e.g., refs. ^2,25^), commenced via several small vents along a 180 m long, north-northeast-trending fracture set in Geldingadalir within the Fagradalsfjall volcanic complex (Fig. 1a). The eruption was preceded by intense seismic unrest in the Fagradalsfjall region, starting on 24th February 2021, which was initially linked to movements on the plate boundary that lies across the RP and possibly involved emplacement of a 5–7 km-long regional dyke between Fagradalsfjall and Keilir^26^. Up until 5th April 2021 the only active vents were 1A and 1B. During this time, venting of magma was characterised by steady bubble-bursting to weakly fountaining activity accompanied by continuous outflow of lava that supplied the bulk of the material initially emplaced in Geldingadalir (Fig. 1b). Five new vents (vents 2–6) opened up along a 1-km-long N30°E-oriented lineament between 5th and 13th April, extending the erupting vent system to the northeast and featuring similar eruptive behaviour to vents 1A and 1B. All except vent 5 ceased to be active towards the end of April. From April to September, vent 5 was the centre of activity and steadily delivered lava to the flow field for >4.5 months via sealed internal pathways (i.e., lava tubes) and surface flows, along with episodic venting of magma through lava fountaining of variable intensity and periodicity (Fig. 2). The eruption produced tephra and a spectrum of lava textural types, including pāhoehoe and ‘a’ā lavas as well as hybrid types (Fig. 2). By the end of September 2021, the eruption had built a volcanic cone rising more than 100 m above the pre-eruption surface and time-averaged magma discharge was fairly steady at about 7 ± 2.5 m^3^/s (DRE; range 2–10 m^3^/s, see http://jardvis.hi.is/eldgos_i_fagradalsfjalli). The main period of sampling for our study was characterised by magma discharge of 6 ± 0.5 m^3^/s (period 19th March–13^th^ April) and 9.8 ± 0.7 m^3^/s (period 13th April–2nd July) (see Supplementary Information for further details). The total lava field covers about 5 km^2^ and has an approximate DRE rock volume of 0.1 km^3^.

### An overview of Fagradalsfjall lavas and magma storage

The lava at Fagradalsfjall is olivine tholeiite and contains ca. 2–15% modal abundances of plagioclase (up to ca. 6 mm in size) and olivine (up to ca. 5 mm in size) set in a finely crystalline to glassy groundmass (Fig. 2). The magma also contains rare clinopyroxene macrocrysts that dominantly exhibit normal zoning, although a small (but significant) portion of these feature reverse, compositional zoning. Plagioclase and olivine macrocrysts feature thin rims with compositions (An_77–80_ and Fo_84–85_, respectively) that are close to being in equilibrium with the erupted melt. Normally zoned plagioclase and olivine macrocrysts feature cores with fairly level An_87–89_ and Fo_87–89_ compositions, respectively. Reversely zoned plagioclase and olivine macrocrysts contain an outer zone of An_87–89_ in plagioclase and Fo_86_ in olivine and a core of An_86–84_ in plagioclase and Fo_84–85_ in olivine (representative mineral data are provided in the Supplementary Datafile). The eruption was preceded by a year-long inflation period and intense seismicity affecting the RP, interpreted as fluids rising from a magma storage reservoir possibly as deep as 15–20 km (ref. ^5^). The geophysical evidence therefore supports a scenario where the eruption drained a lower crustal magma storage reservoir, as is typical for Icelandic rift zones^27^. This realisation permits us to investigate the sources of the melts feeding this storage zone and to constrain how the composition of magmas supplying the eruption changed over time.

### Major and trace element results

**Fig. 3:**
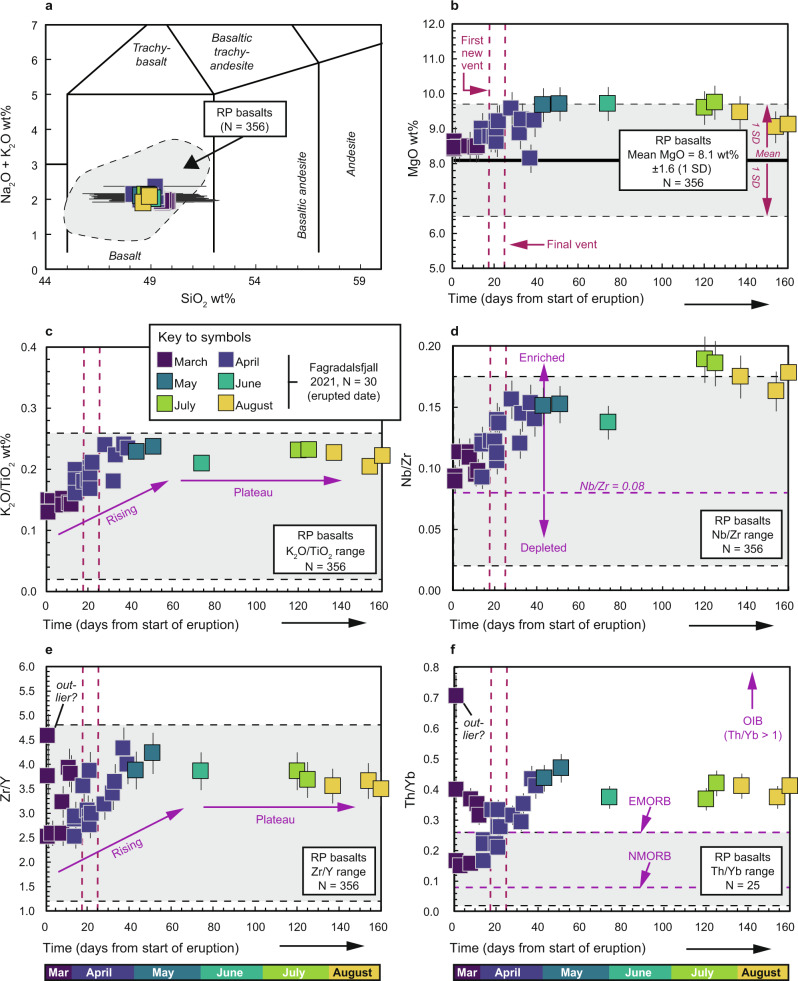
Temporal changes in geochemistry of Fagradalsfjall whole-rocks. **a** TAS diagram after ref. ^49^ with comparative literature data for Reykjanes Peninsula (RP) basalts. Fagradalsfjall whole-rock lava samples are tholeiitic basalts. **b**–**f** Timeline plots of MgO (**b**), K_2_O/TiO_2_ (**c**), Nb/Zr (**d**), Zr/Y (**e**), and Th/Yb (**f**). In **b**, the mean MgO wt.% for RP basalts is shown as a black horizontal line and the height of the grey box corresponds ±1 SD; for all other plots the full range of literature data is shown. In panel **d**, enriched samples are defined as having a Nb/Zr ratio >0.08 (after ref. ^28^). In panel **f**, mantle end-members are indicated after ref. ^50^. Note that there are relatively few Th/Yb ratios available for RP basalts in the literature. The dashed vertical lines in all panels indicate the timing of additional vent openings; the second vent (#2) and the last new vent (#6) as labelled in panel **b**. Abbreviations: EMORB enriched mid-ocean ridge basalt, NMORB normal mid-ocean ridge basalt, OIB ocean island basalt, RP Reykjanes Peninsula. Uncertainty on major and trace element data is estimated at better than 5% and 10%, respectively (shown as error bars). Where no error bars are visible, uncertainty is smaller than symbol size. Literature data are taken from refs. ^25,28,51–56^.

**Fig. 4:**
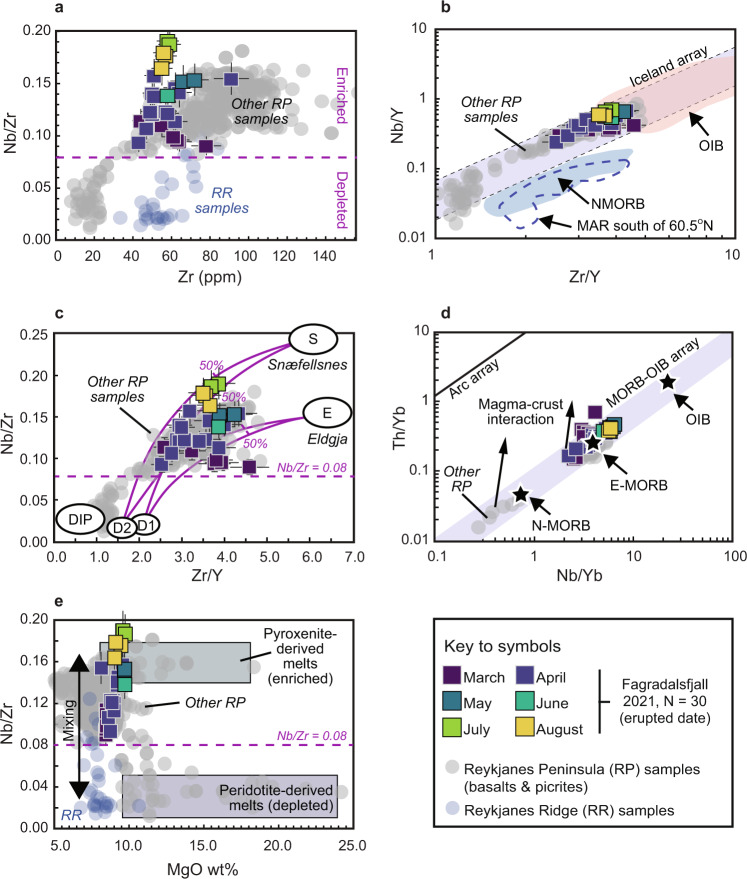
Trace element ratios in whole-rock samples from the 2021 Fagradalsfjall eruption. **a** Zr versus Nb/Zr diagram after ref. ^25^ showing that early erupted samples are less enriched than later erupted ones. Fagradalsfjall samples are notably distinct from Reykjanes Ridge (RR) basalts reported in ref. ^57^. **b** Zr/Y versus Nb/Y diagram showing that while Fagradalsfjall samples plot within the Iceland array, they are compositionally distinct from NMORB and Mid-Atlantic Ridge (MAR) south of 60.5°N (reference fields drawn after ref. ^58^). **c** Zr/Y versus Nb/Zr diagram showing the Depleted Iceland Plume component (DIP), two depleted mantle components (D1 and D2), the main basaltic component (“E” for Eldgjá), the alkalic component (“S” for Snæfellsnes), and mixing curves after ref. ^10^. The samples from the 2021 Fagradalsfjall eruption reflect an apparently co-equal mixture of an enriched mantle component and a more depleted mantle endmember in magma genesis. **d** Nb/Yb versus Th/Yb diagram after ref. ^50^ showing that Fagradalsfjall samples plot dominantly around E-MORB. **e** MgO versus Nb/Zr diagram after ref. ^59^ showing enriched (pyroxenite) and depleted (peridotite) endmember mantle sources after ref. ^60^. Samples that plot between these end-members reflect melt mixing and concurrent crystallisation. Abbreviations and data uncertainties are given in Fig. 3. Sources of literature data are the same as in Fig. 3 with additional data from ref. ^57^.

We analysed 30 lava and tephra samples for bulk-rock major and trace elements. All major element data were normalised on a volatile-free basis before plotting and the normalised values are reported here (sample coordinates and descriptions are provided in the Supplementary Information and geochemical data are provided as a Supplementary Datafile). A total alkali versus silica (TAS) diagram classifying the samples as sub-alkaline basalts is shown in Fig. 3a. Selected major elements and trace element ratios are plotted versus time in Fig. 3 and trace element ratio plots are shown in Fig. 4. All of the samples have low loss on ignition (LOI) values (see Supplementary Datafile) and they show a narrow range in SiO_2_ from 48.1 to 49.8 wt.% and a somewhat wider range in MgO from 8.1 to 9.7 wt.%. Notably, both the MgO content and the K_2_O/TiO_2_ ratio of the samples increased steadily during the first two months of the eruption before plateauing from May onwards (Fig. 3b, c). Other major element oxides do not show significant variations over time (e.g., SiO_2_, Na_2_O, CaO, Al_2_O_3_, and Fe_2_O_3_(total); Fig. S2). Trace element ratios Nb/Zr, Zr/Y, and Th/Yb all show a marked increase over time, with overall ranges of 0.1 to 0.2, 2.5 to 4.6, and 0.2 to 0.7, respectively (Fig. 3d–f). This variation is outside the range of analytical uncertainty and is unlikely to be due to different crystal modal abundances in the analysed samples, since the overall macrocryst modes are relatively restricted at 2–15% and the samples do not display large macroscopic differences. Overall, the compositions of the new Fagradalsfjall samples overlap with previously reported data for RP basalts (see Fig. 3). Ratios of Th/Yb only partly overlap with the existing data, but this may be because there are relatively few Th/Yb ratios available for RP basalts (Fig. 3f). Importantly, as illustrated in Fig. 4a, where our samples are shown on a Zr versus Nb/Zr plot, it can be seen that all of the analysed Fagradalsfjall samples are enriched, based on a Nb/Zr value >0.08 (after ref. ^28^). In Fig. 4b–d, we compare our samples to various mantle components such as Ocean Island Basalt (OIB) and Mid-Ocean Ridge Basalt (MORB), as discussed further below.

### Oxygen and hydrogen isotope results

**Fig. 5:**
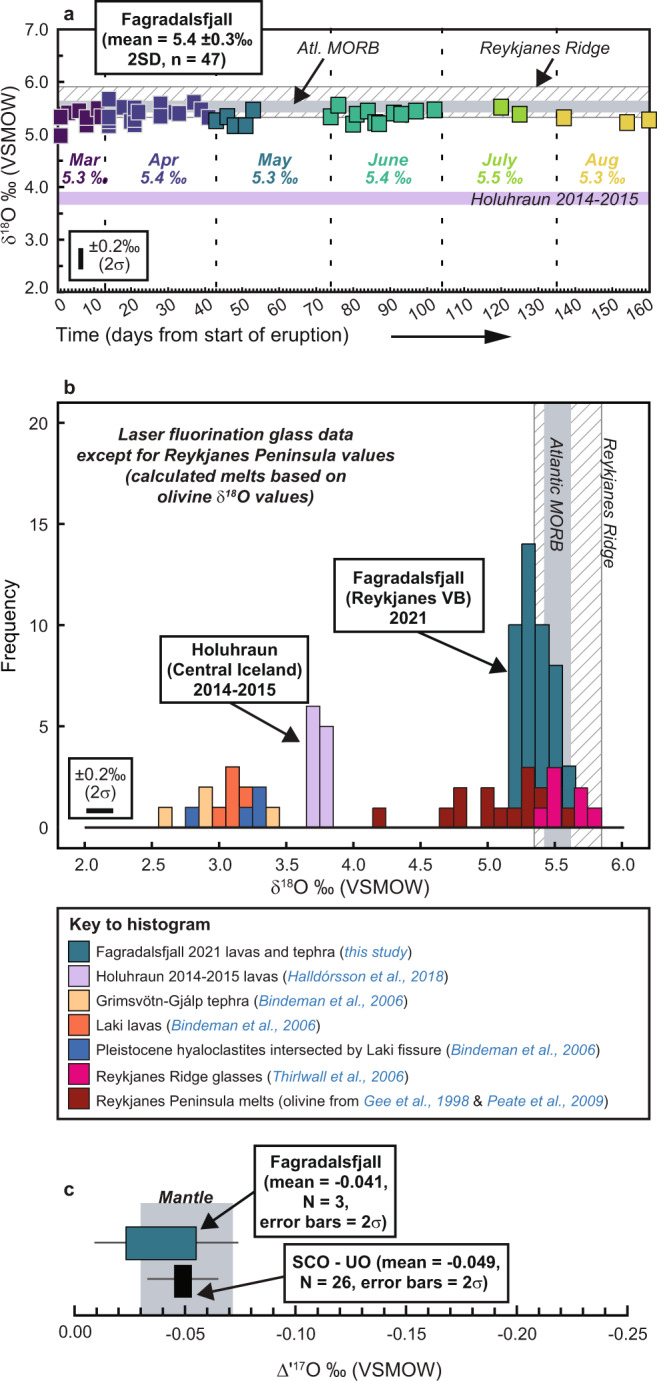
Oxygen isotope results. **a** Timeline of δ^18^O values obtained for glasses from the 2021 Fagradalsfjall eruption over the period 20th March to 26th August 2021. Analytical uncertainty is displayed as an inset. A total of 47 analyses are presented (repeat analyses were made on four samples; in these cases the mean value is plotted), which yielded an overall mean of 5.4 (±0.3‰ 2 SD). The mean δ^18^O value for each month is also shown in italics. The range of values from the previous Icelandic eruption at Holuhraun (Central Iceland) in 2014–15^21^ is provided for comparison. Note that the Fagradalsfjall data overlap Atlantic MORB^22^ and Reykjanes Ridge glasses^19^. **b** Frequency distribution plot (frequency = number of samples) comparing the new 2021 Fagradalsfjall eruption data to glasses from the 2014–15 Holuhraun eruption^21^, Grímsvötn-Gjálp^17^, the 1783–4 Laki eruption^17^, and the Reykjanes Ridge^19^. Equilibrium melt δ^18^O values were calculated from Reykjanes Peninsula olivine data in refs. ^25,28^ using fractionation factors calculated after ref. ^61^. Bin size is 0.1‰, equivalent to 1σ uncertainty on the data. **c** Δ′^17^O values obtained for three Fagradalsfjall samples compared to values obtained for the San Carlos olivine (SCO) standard at the University of Oregon (UO) and the mantle range^37^. Abbreviations: Apr April, Atl Atlantic, Aug August, Mar March, MORB Mid-Ocean Ridge Basalt, Reykjanes VB Reykjanes Volcanic Belt.

**Fig. 6:**
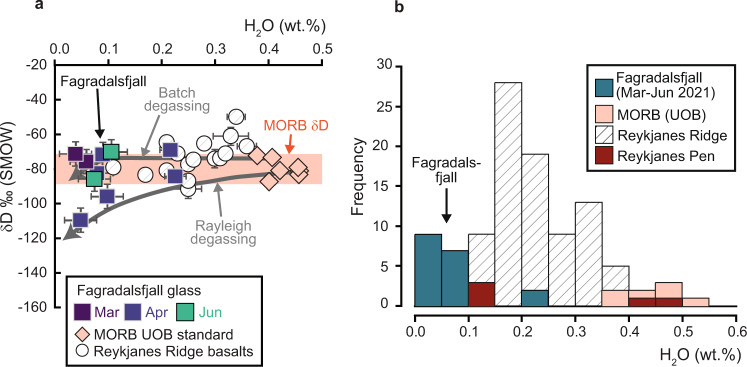
Hydrogen isotope results. **a** δD-H_2_O variation in glasses from the 2021 Fagradalsfjall eruption compared to the MORB UOB (University of Oregon Basalt) glass standard^30^. The δD range for fresh MORB is drawn after ref. ^29^. The mild decrease in δD observed in some of the Fagradalsfjall samples is likely due to near-surface degassing (Batch and Rayleigh degassing curves shown from a starting composition similar to UOB). Reference data for Reykjanes Ridge basalts are plotted after ref. ^62^. Analytical uncertainty decreases with increasing water content; error bars are displayed for low water samples and are otherwise smaller than symbol size. **b** Frequency distribution plot of H_2_O contents in Fagradalsfjall glasses, including samples with low water contents (0.00–0.03 wt.% H_2_O) that have no accompanying δD values. There is no evidence for magma interaction with seawater or meteoric water that would hydrate the glass and cause an increase and decrease in H_2_O contents and δD values, respectively. Additional H_2_O data for Reykjanes Ridge and Reykjanes Peninsula basalts are plotted after ref. ^41^. Abbreviations as before.

We analysed 47 glassy samples for oxygen isotopes (δ^18^O values), as shown in Fig. 5a, b. In addition, three samples were analysed for Δ′^17^O (Fig. 5c) and ten analyses were made for H_2_O contents and hydrogen isotopes (δD values; Fig. 6). The δ^18^O values of our samples range from 5.0 to 5.7‰, with an overall mean of 5.4‰ (±0.3 2 SD, *N* = 47). Considering the data on a month-by-month basis, the mean δ^18^O value for March, April, May, June, July, and August is 5.3, 5.4, 5.3, 5.4, 5.5, and 5.3‰, respectively (the total number of samples for each month is 9 for March, 17 for April, 5 for May, 11 for June, 2 for July, and 3 for August). Given that the natural variability on the oxygen isotope data of glass is 0.2‰ (95% confidence interval; the analytical precision is better than 0.1‰), the monthly mean δ^18^O values for Fagradalsfjall are indistinguishable. The δ^18^O values obtained here furthermore overlap within analytical uncertainty with values reported for MORB (average MORB value of 5.5‰^22^) and, importantly, Fagradalsfjall samples with diverse trace element ratios have identical δ^18^O values. Regarding Δ′^17^O, the values presented here are among the first reported for fresh Icelandic basalts and range from −0.031 to −0.048 ± 0.012‰. These values overlap the mantle range (Fig. 5c), and are thus in good agreement with the δ^18^O values outlined above.

The δD values for our Fagradalsfjall samples range from −69.2 to −109.7‰ and yield a mean of −81.8 ± 26‰ (2SD, *N* = 10). Except for the most degassed sample (δD = −109.7‰), these values overlap within analytical uncertainty with the canonical range for pristine MORB of −80 ± 10‰ (see ref. ^29^) but are ca. 10‰ higher than North Atlantic depleted MORB^29^. We furthermore performed eight new analyses of MORB basaltic reference material (University of Oregon Basalt, “UOB”, an EMORB from the East Pacific Rise^30^), which was run concurrently with the Icelandic unknowns and yielded δD values ranging from −72.2 to −87.3‰ with a mean of −79.2 ± 10‰ (2 SD, *N* = 8). While performing the hydrogen isotope analyses, we also performed 17 water analyses on Fagradalsfjall glass samples. However, several of these samples were anhydrous, containing less than ~0.03 wt.% water, which was not detectable by the High Temperature Conversion Element Analyzer employed here (TC/EA, see Methods) and thus these samples have no accompanying δD value. The H_2_O contents of our hydrous Fagradalsfjall glasses range from 0.00 to 0.23 wt.% and the UOB MORB reference glasses analysed here have H_2_O contents ranging from 0.38 to 0.53 wt.%. The subaerial and partly degassed Fagradalsfjall samples are thus less hydrous than the submarine MORB glass standard (UOB), which was quenched at ocean floor depths.

## Discussion

The samples from the 2021 Fagradalsfjall eruption are geochemically similar to earlier erupted basalts on the RP (Figs. 3–4). Looking at the geochemistry timeline plots (Fig. 3), it appears that the earliest eruption products are compositionally more heterogeneous with respect to their trace element contents than the later products (assuming no analytical artefacts or significant variations in crystal modal abundances, as outlined above). This pattern is well demonstrated in Fig. 3 where it can be seen that Zr/Y (Fig. 3e) and Th/Yb (Fig. 3f) show significant variability in lavas erupted during the first phase of the eruption when only vents 1A and 1B were active and then level out after the opening of vent 6. Similarly, MgO and K_2_O/TiO_2_ ratios increase early in the eruption and then level out somewhat over time. However, it is striking that Nb/Zr values increase from the eruption onset all the way to late August 2021, some 160 days after the eruption start date (Fig. 3d). These geochemical variations probably reflect a dynamic magma reservoir, which was likely chemically zoned, repeatedly fed by mafic magma replenishments, and composed of highly variable melts sourced from a heterogeneous mantle. We lay out our reasoning for this assessment below.

Four hypotheses can be put forward to explain the compositional variations observed in the Fagradalsfjall major and trace element data: (i) Fractional crystallisation of a single basalt parent magma; (ii) Crustal assimilation modifying a basaltic parent magma (e.g., by assimilation and fractional crystallisation; AFC); (iii) Different mantle melting degrees with a single source, but displaying variations in the depth/temperature of melting; and (iv) Involvement of closely spaced and simultaneously tapped multiple mantle sources/endmember compositions such as depleted or variably enriched peridotite-pyroxenite plume components as previously suggested to reside under Iceland.

Hypothesis (i) (fractional crystallisation) can be excluded as the principal cause for the large variation of incompatible trace elements observed here, because the studied samples do not display systematic major element variations characteristic of fractional crystallisation of olivine + plagioclase ± clinopyroxene (see Fig. S2). Geophysical evidence moreover suggests that this eruption was fed from lower crustal, near-Moho depths at ca. 15–20 km (ref. ^5^), which is consistent with magma storage in a lower crustal reservoir (cf. ref. ^27^) as opposed to at shallower levels where protracted crystal fractionation (and crustal assimilation, as discussed below) would be expected to occur.

Hypothesis (ii) (crustal assimilation) can also be excluded as the dominant cause of the observed temporal variations because of the essentially homogeneous oxygen isotope composition of the Fagradalsfjall lavas. The crust under Reykjanes is highly diverse with respect to its oxygen isotope composition and even more so with respect to hydrogen isotopes (refs. ^31,32^) as evidenced by hydrothermally altered rocks in deep drill holes at the tip of Reykjanes. Oxygen isotopes are sensitive to incorporation of even small amounts of hydrothermally altered, low-δ^18^O and low-δD crustal materials, which in Iceland can differ from mantle-derived materials by several permille (‰) (e.g., see the plotted hyaloclastite δ^18^O values in Fig. 5b and ref. ^17^). As both oxygen and hydrogen isotopes point to typical MORB-like composition for Fagradalsfjall (Figs. 5–6), we consider that petrogenesis at Fagradalsfjall is very different from the situation further eastwards in Iceland such as at Grímsvötn^17^, Holuhraun^21^, or Askja^20^, where lavas and primitive melt inclusions have been found to possess relatively low δ^18^O values (e.g., recent lavas at Holuhraun have a mean δ^18^O value of 3.8‰ (*N* = 11 samples; ref. ^21^; see also Fig. 5), which have been discussed in the context of magmatic assimilation of low-δ^18^O hydrothermally altered crust. However, we do consider that localised crustal assimilation is possible at Fagradalsfjall, e.g., at the edge of the long-operating fissure that would bring the temperature of the contact zone closer to that of the flowing magma. This process might have influenced individual outliers in our sample suite, but was likely a minor process overall.

Similarly, hypothesis (iii) (mantle melting variation of a single source) is unlikely, since the degree of melting would need to vary by a factor of two or more to fully explain the observed variations of incompatible trace elements by variations in mantle melting degree alone. This is because such variation in melting degree should leave ratios of similarly incompatible trace elements, such as K/Ti, Zr/Nb relatively invariant, which is not what is observed in our temporal dataset (e.g., Figs. 3–4), although it would produce little effects on δ^18^O and δD values (e.g., ref. ^33^).

Thus, hypothesis (iv) (melting of diverse mantle types) appears to provide the best explanation for the variations we observe in our data. In terms of their incompatible trace element ratios, Fagradalsfjall basalts are similar to previously published enriched samples from the Reykjanes Peninsula and they are distinct from depleted Reykjanes Ridge samples (Fig. 4a). Fagradalsfjall basalts are also distinct from NMORB, instead plotting close to more enriched mantle end-members such as E-MORB and OIB (i.e., Snæfellsness and Eldgjá end-members), with the magma tapped from the lower crustal reservoir becoming seemingly more enriched over time (Fig. 4). Such a multi-component source is a useful first order approximation to explain the Fagradalsfjall data. As trace element ratios of Zr/Y, Nb/Zr, and Nb/Y (Fig. 4) are relatively insensitive to fractionation processes in mafic magmatic systems they are interpreted here to reflect variable mantle source components and would thus be reflective of Icelandic plume materials (i.e., ancient recycled E-MORB and OIB in addition to pyroxenitic and peridotitic source regions) feeding the lower crustal magma storage reservoir beneath Fagradalsfjall. Remarkably, in terms of their trace element ratios, the Fagradalsfjall basalts span roughly half of the previously described range for Reykjanes Peninsula basalts (Fig. 4), supporting their derivation from a heterogeneous mantle source. The variable enrichment of incompatible trace elements across Iceland has previously been considered to reflect an inhomogeneous plume (e.g., refs. ^7,10,34,35^) with variable mixtures of an N-MORB envelope and a plume of E-MORB type mantle and enriched (OIB) plume centre. Murton et al. (ref. ^10^) offered correlation diagrams employing incompatible trace element ratios (Nb/Zr vs Zr/Y) that require mixing of at least three components: the low trace element ratio components characteristic of the Reykjanes Ridge (D1 and D2) and an enriched source (E1). As can be seen in Fig. 4c, Fagradalsfjall samples fall within a region of compositional space that is defined by the D1/D2 endmember and the enriched Eldgjá (E) and alkalic Snæfellsnes (S) endmembers, which argues for a multi-component mixing array. The involved mantle components are thus distinct from a strongly enriched Iceland plume centre-type basalt as well as from an Atlantic N-MORB source and are best explained as reflecting a heterogeneous mantle that is part of the slightly enriched fringe surrounding the more enriched plume centre (cf. refs. ^7–10^). Interpreting our data in the context of, e.g., Fitton et al. and Kempton et al. (refs. ^7,9^), who suggested that the mantle beneath the southern Reykjanes Ridge is E-MORB-like, and Murton et al. (ref. ^10^), who suggested that the Eldgjá eruptives (E) are representative of the enriched plume, the 2021 Fagradalsfjall eruption may have been fed from what previous workers termed the Icelandic “plume sheath”. This sheath possibly envelopes a more strongly enriched plume centre and may be compositionally zoned (e.g., refs. ^7,9,10^). We also note that enriched mantle domains have been shown to retain higher melt fractions than more depleted domains as they cool^36^, which might in part explain the increase in the enriched component over time at Fagradalsfjall.

The δ^18^O values obtained for Fagradalsfjall basalts are remarkably homogeneous over the eruption lifetime (mean = 5.4 ± 0.3‰ from March to August 2021, which covers almost the entire eruption given that activity ceased in mid-September 2021; Fig. 5a), despite variable trace element ratios that indicate the involvement of multiple distinct mantle sources in magma genesis. The Fagradalsfjall δ^18^O values moreover overlap within analytical uncertainty with the average value for global MORB of 5.5 ± 0.1‰ reported by ref. ^22^ and, similarly, their Δ′^17^O values overlap the reported mantle range^37^ (Fig. 5c). The recent reference values for the δ^18^O composition of MORB^22^ were established by employing a large number of analyses obtained using modern techniques (laser fluorination of glass) and are thus considered more accurate than previous estimates for MORB of ca. 5.7 ± 0.3‰ (e.g., ref. ^38^). Older estimates relied on bulk analyses using conventional fluorination of powdered glass samples, an approach, which has now been superseded by modern techniques for analysing oxygen isotopes in silicate materials (see also discussion in ref. ^39^). In this respect, it is particularly noteworthy that the new Fagradalsfjall δ^18^O values are significantly different to values obtained from Central Iceland, some of which were obtained using the same analytical techniques as those employed here (laser fluorination). This difference is exemplified by the 2014–2015 Holuhraun eruption, which yielded mean δ^18^O values of 3.8‰ (*N* = 11 samples; ref. ^21^), making Holuhraun basalts on average 1.6‰ isotopically lighter than Fagradalsfjall basalts (Fig. 5b). A key observation from our work is that despite the fact that Fagradalsfjall basalts cover roughly half the entire recorded trace element variability for the RP, their δ^18^O values are homogenous and similar to “normal” MORB, being perhaps only mildly (0.1–0.2‰) more depleted than average global MORB^22^. Therefore, despite there being evidence for multiple distinct mantle components involved in Fagradalsfjall magma genesis, our data support the absence of ^18^O-depleted mantle supplying the Reykjanes Peninsula. This contrasts with studies focussed on other parts of Iceland, such as Theistareykir (Northern Volcanic Zone), where the mantle source has been discussed as having variable and low δ^18^O values^23,40^. We offer the suggestion that if the oxygen isotope values presented in this study are representative of various Icelandic plume components, then that would imply that the mantle underlying Iceland has a relatively uniform primary δ^18^O value. If this is indeed the case, then a follow-on implication might be that high-Mg basaltic lavas with low δ^18^O values at, e.g., Holuhraun are the result of assimilation of low δ^18^O crust or of delamination of low δ^18^O crust into the mantle source. These regional aspects are, however, speculative and somewhat beyond the scope of the current work but they may be an attractive target for future studies.

With respect to the volatile content and hydrogen isotope composition of Fagradalsfjall lavas, most of the samples analysed here possess δD values that overlap within uncertainty with the range for canonical global MORB of −80 ± 10‰, but are slightly higher than North Atlantic depleted MORB and lower than North Atlantic Enriched MORB (see ref. ^29^). The samples are also overall less hydrous than both MORB and Reykjanes Ridge samples^41^ (Fig. 6). The expected primary water content for a MORB or EMORB-derived basalt is 0.2 to 0.6 wt.% (e.g., refs. ^29,42^), but degassing leads to a decrease in water content and δD value. If degassing from a primary magma with 0.6 wt.% H_2_O is incremental and of Rayleigh type, it can result in a change of up to 20–30‰ in the δD value of the residual basaltic glass^43^, but only a change of 10‰ would be expected if the degassing process is of Batch-type^44^. We modelled both types of degassing employing starting compositions similar to the UOB MORB glass standard (0.40 to 0.45 wt% H_2_O; Fig. 6) and conclude that both Rayleigh and Batch degassing probably occurred during the Fagradalsfjall eruption.

**Fig. 7:**
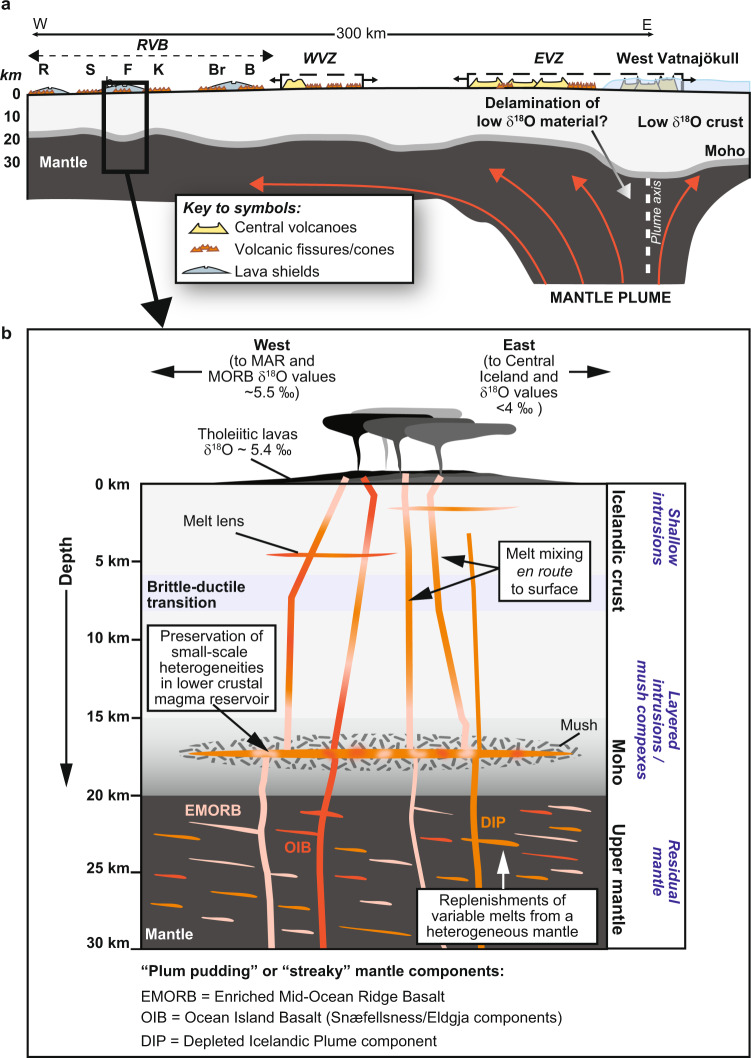
Schematic model. **a** Sketch placing Fagradalsfjall (F) in a regional context in relation to the Reykjanes Volcanic Belt (RVB), the Western Volcanic Zone (WVZ), and the Eastern Volcanic Zone (EVZ) of Iceland (see Fig. 1 for map view of Iceland). **b** Close-up of Fagradalsfjall showing a heterogeneous “plum pudding” or “streaky” mantle comprising various source components such as EMORB, OIB, and DIP. Variable melts from this heterogeneous mantle were assembled in a lower crustal magma reservoir, which likely experienced multiple replenishment episodes leading to complex mineral zoning. Mixing of melts shortly before eruption facilitated preservation of compositional heterogeneities inherited from the mantle source. Remarkably, despite the variability in the compositions of Fagradalsfjall erupted products, their δ^18^O values are homogeneous at 5.4‰ on average with no evidence for low-δ^18^O mantle or interaction with low-δ^18^O crust. The mean δ^18^O value for Fagradalsfjall may thus represent a useful mantle reference in this part of the Iceland plume system. Abbreviations: B Bláfjöll, Br Brennisteinsfjöll, K Krýsuvík, MAR Mid-Atlantic Ridge, MORB Mid-Ocean Ridge Basalt, R Reykjanes, S Svartengi.

Here, we put forth a tentative model regarding the nature of closely spaced mantle components under the Reykjanes Peninsula that fed the Fagradalsfjall eruption (cf. refs. ^7,9,10^). The first proposed idea involves discrete domains (“streaks” in the context of a streaky or plum-pudding type mantle models) of more fertile, perhaps pyroxenitic mantle, dispersed in an otherwise less enriched (E-MORB) Icelandic plume matrix (see also Fig. 4e). The second proposed idea involves a plume with a strong lateral variation in the degree of enrichment and, additionally, differences in the degree of melting with depth. The latter implies the need for significant lateral magma flow to the same location of the magma exit channel. Irrespective of the detailed geometrical arrangement of the variously enriched source components required to feed the recent Fagradalsfjall eruption, a mixture of depleted mantle, E-MORB, and more enriched Icelandic (OIB) plume components (i.e., Eldgjá and Snæfellsnes) are the main sources required to explain the data and must therefore be assumed to be the major mantle components under the Reykjanes Peninsula (Fig. 7). With respect to its volatile load, the Fagradalsfjall magma likely possessed MORB-like water contents (estimated here at around 0.45 wt.% H_2_O), but much of this water was lost during near-surface degassing, a process which also modified the lava δD values (Fig. 6). Importantly, despite the different mantle source compositions involved, the Fagradalsfjall lava δ^18^O values are remarkably homogenous and indistinguishable from “normal” MORB. The lavas also possess Δ′^17^O and δD values overlapping with the mantle range. The mantle-like and invariant oxygen isotope ratios reported here for Fagradalsfjall lavas are likely the result of direct magma ascent from near-Moho levels after only a short residence time (perhaps tens to hundreds of years) in a lower crustal storage reservoir at ca.15–20 km depth (Fig. 7). This would allow for preservation of original δ^18^O mantle signals, since in this scenario there would be insufficient time for magma-crust interaction processes to modify original δ^18^O values. Our data therefore lead us to suggest that the mantle supplying magmatism along the Reykjanes Peninsula has MORB-like δ^18^O values and that there is an absence of low δ^18^O source components under this part of Iceland. While additional work would be required to assess if this finding is applicable further afield in Iceland, the Fagradalsfjall case nonetheless represents a useful mantle δ^18^O reference value in this part of the Icelandic plume system.

## Methods

### Oxygen isotopes (δ^18^O and Δ′^17^O)

All oxygen isotopic data in this study were obtained at the University of Oregon (UO), Eugene, Oregon, USA. Detailed method descriptions can be found in refs. ^37,45,46^ and are summarised here. Oxygen isotope measurements for δ^18^O utilised 1 to 1.5 mg of glassy sample material and were performed by laser fluorination and gas-source mass spectrometry (MAT253). Purified BrF_5_ was used as a reagent and a boiling mercury diffusion pump was used to remove excess F_2_ gas remaining after reaction of BrF_5_ during laser fluorination. Purified O_2_ was converted into CO_2_ and then run in dual inlet mode on a MAT253 mass spectrometer integrated with a vacuum line. San Carlos Olivine (δ^18^O = 5.25‰), UWG2 garnet (δ^18^O = 5.80‰) and an indoor UOG garnet standard calibrated relative to the other two (δ^18^O = 6.52‰) were used to calibrate the data on the VSMOW scale. Each session included analyses of four to six standards and correction for day-to-day variability was 0.0 to 0.2‰. Reference CO_2_ gas was used as a working standard and it was periodically rerun against OZTECH CO_2_ gas. Standard deviations on standards in individual sessions ranged from ±0.00 to ±0.11‰ (±0.06‰ on average; one standard deviation). Samples from sessions with less precise standard data were rerun and the average was used for plotting purposes with the full duplicates/triplicates reported in the Supplementary Datafile. For triple oxygen isotope analyses, the generated gas was run as O_2_ along with the SCO standard (Δ′^17^O = −0.052‰) and a triple O continuous flow line specially constructed for precise triple O measurements was utilised. The generated O_2_ gas was run through an 8 ft long gas chromatographic column at room temperature for purification of NFx compounds. Generated gases were additionally frozen on a 5 Å zeolite sieve and then released into the bellow of the mass spectrometer by an LN_2_-ethanol mix over 10 min. GC and zeolite traps were degassed with flowing He at 200 °C for 15–20 min between each sample. The purified O_2_ gas was run five times with eight cycles in dual inlet mode against a well calibrated oxygen gas standard on a MAT253 isotope ratio mass spectrometer. For triple oxygen isotope analyses of the Icelandic samples reported in this study, four concurrently run UWG standards were used to correct data to the VSMOW scale. Additionally, a set of 26 olivine grains from mantle nodules (quoted as average) run before this session provided another point of calibration for the obtained Icelandic dataset with that of the mantle. Our indoor San Carlos Olivine has the following values: δ^18^O = 5.25‰ and Δ′^17^O_0.5305_ = −0.052‰.

### Hydrogen isotopes and water measurements

Hydrogen isotopes were measured on groundmass glass (50–200 µm size fraction) using a TCEA-MAT-253 system at the University of Oregon. Methods are after refs. ^30,47^ and are summarised here. Samples weighing between 8 and 18 mg were loaded into Ag packets and dropped into a glassy carbon reactor held at 1450 °C. Water was quantitatively reduced into H_2_ and CO gases, which were separated by a GC column. H_2_ was carried over to the open split in front of the mass spectrometer by He flow, and analysis included 3 reference gas peaks and a single unknown gas peak. D/H ratios and total water in glass were determined using concurrently run standards of USGS57 and 58 micas and a MORB glass standard (UOB). Water concentration was detected by peak integration if its content was greater than ~0.02–0.04 wt.%; precision on D/H at water concentration <0.1 wt.% was ±8 ‰ and at higher concentration it was ±5‰.

### Electron probe microanalysis (EPMA)

Groundmass glasses and minerals in tephra and quenched lava samples were analysed at the University of Iceland on a JEOL JXA-8230 electron probe microanalyzer (EPMA) using an acceleration voltage of 15 kV, beam current of 10 nA and beam diameter of 10 μm. The international A99 standard was used to monitor for instrumental drift and maintain consistency between measurements.

### Major and trace elements

Whole-rock sample powders were analysed for major and trace elements at Activation Laboratories Ltd., Ancaster, Canada using their research grade package 4LITHORES. After fusion with lithium metaborate/tetraborate and digestion in nitric acid, major elements were measured by inductively coupled plasma optical emission spectrometry (ICP-OES) and trace elements were analysed by inductively coupled plasma mass spectrometry (ICP-MS) on a Perkin Elmer Sciex ELAN 6000, 6100, or 9000 ICP-MS. Detection limits are 0.01 wt.% for all major element oxides, except for MnO and TiO, which have detection limits of 0.001 wt.%. Detection limits for trace elements range from 0.01 to 30 ppm. Data quality was verified by repeated analysis of internal reference materials and analytical precision is estimated at better than 5% for major elements and better than 10% for trace elements. One sample of Fagradalsfjall basalt was analysed in duplicate; the two sets of analyses are virtually identical. Information about detection limits for each element as well as the results of standard analyses and the Fagradalsfjall duplicate are provided in the Supplementary Datafile.

## Supplementary information

Supplementary Information

## Data Availability

The authors declare that all data generated in this study are provided in the Supplementary Information and Source Data file. Source data are provided with this paper.

## Source data

Source Data
