# Supplementary material for: Diverse mantle components with invariant oxygen isotopes in the 2021 Fagradalsfjall eruption, Iceland
Source: Nat Commun. 2022 Jun 29;13:3737. doi: 10.1038/s41467-022-31348-7 (PMC9243117; doi:10.1038/s41467-022-31348-7)
Supplement: Supplementary file 1 — Supplementary Information [file 41467_2022_31348_MOESM1_ESM.pdf]

Diverse mantle components with invariant oxygen isotopes in the 2021 Fagradalsfjall eruption, Iceland

I.N. Bindeman, F.M. Deegan*, V.R. Troll, T. Thordarson, A. Höskuldsson, W. Moreland, E.U. Zorn, A.V. Shevchenko, T. R. Walter

Supplementary Information

Contents:

Supplementary Figure 1: Eruption periods on the Reykjanes Peninsula over the last 4000 years.

Supplementary Figure 2: Major element variations in Fagradalsfjall samples.

Supplementary text and Figures S3 – S4: Photogrammetric analysis of eruption site.

Supplementary references.

Supplementary Table 1: Sample information.

Geochemical data are available as a separate datafile.

*Correspondence to: frances.deegan@geo.uu.se

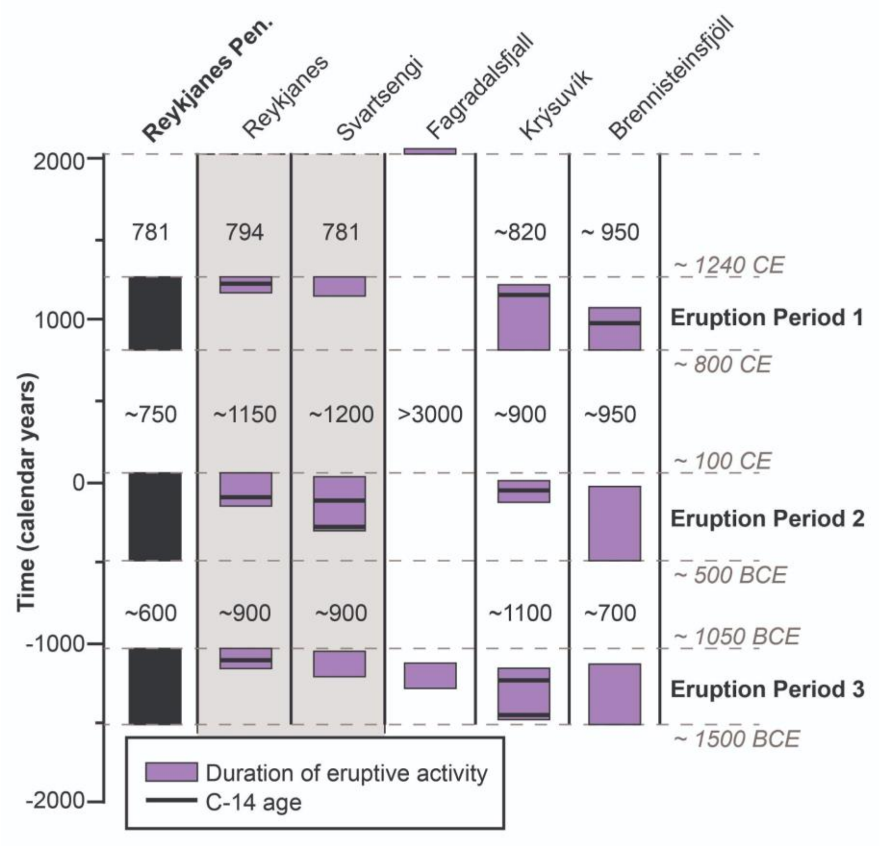

Fig. S1. Eruption periods on the Reykjanes Peninsula (RP) over the last 4000 years. The time scale is in calendar years (CE = Common Era; BCE = Before Common Era). The purple bars indicate the extent of the eruption activity within individual systems, while the extent of each eruption period on the RP is indicated by black bars. The length of each repose period is indicated by the figures between the bars. Carbon-14 age determinations are indicated by black horizontal lines. The Reykjanes and Svartsengi volcanic systems are shaded grey to indicate that by some researchers they are considered to represent a single system. Modified after the ISOR online geological map of Iceland (jardfraedikort.is) and ref.[1].

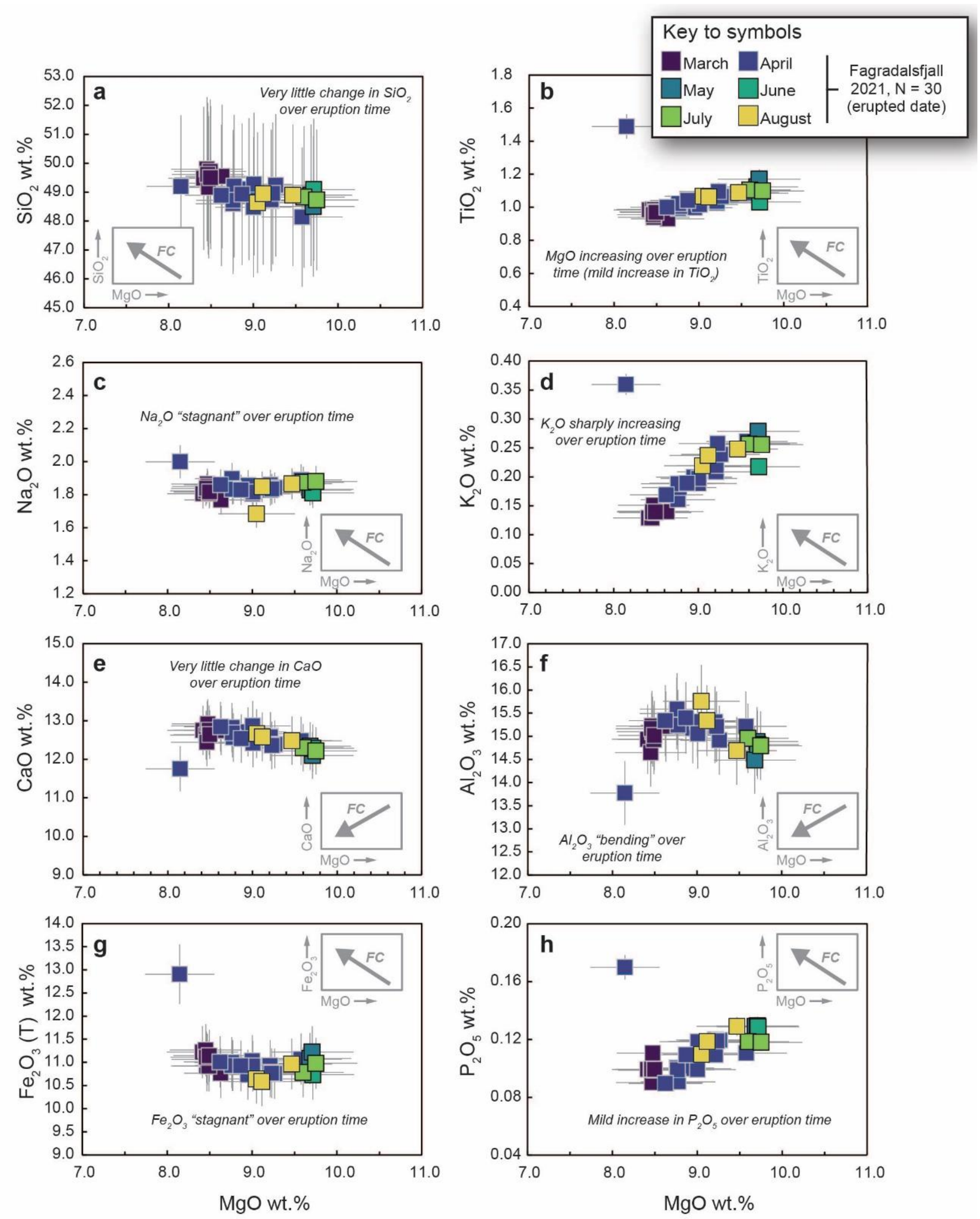

Fig. S2. Major element variations in Fagradalsfjall samples. Major element oxides (in wt.%) of SiO_2 (**a**), TiO_2 (**b**), Na_2O (**c**), K_2O (**d**), CaO (**e**), Al_2O_3 (**f**), Fe_2O_3 total (**g**), and P_2O_5 (**h**) plotted against MgO for Fagradalsfjall samples erupted between March and August 2021. Insets show expected trends for fractional crystallisation (FC) over the MgO range of 7 to 11 wt.%, based on trends for Reykjanes Ridge basalts in ref.[2]. Note that the Fagradalsfjall data do not show systematic variations consistent with FC and most major elements show trends at odds with FC. All data normalized to 100%.

Supplementary text: photogrammetric analysis

In order to calculate lava volumes and discharge rates during the 2021 Fagradalsfjall eruption, we performed photogrammetric analysis of optical aerial and satellite data. The aerial data were collected on the 13th of April 2021 with Mavic Pro 2 UAV at a flying height of 120 m above the starting point. The satellite data were tri-stereo panchromatic images acquired on the 2nd of July 2021 with PHR1A Pleiades sensor. We processed the data using Agisoft Metashape v. 1.5.2 software and obtained ~5-cm-resolution UAV point cloud and ~2-m-resolution Pleiades point cloud. The relative errors of the point clouds construction don't exceed 0.3 m. The absolute accuracy of the point clouds was corrected by aligning to the pre-eruption DEM created by the National Land Survey of Iceland LMI (http://atlas.lmi.is/mapview/?application=DEM). The pre-eruption topography currently (2022) can be available as an identical 2-m-resolution DEM (ArcticDEM) (https://www.arcgis.com/apps/webappviewer/index.html?id=aff5fa8f5d5548c6bff44cc8be385f61). The alignment was performed manually using CloudCompare v. 2.9.1 software. The alignment errors were calculated by comparing Z distances (from 0 to 2.2 m) between two point clouds measured at several points picked at different areas not affected by the eruption but close to the lava field. Using the extracted Z differences, we calculated the final root mean square errors (RMSEs) of the point clouds alignment.

Comparison with the pre-eruption DEM (Fig. S3, S4) let us calculate volumetric changes between the point clouds and estimate magma discharge rates. The obtained RMSEs were distributed over the areas of the lava field to estimate the volume errors (from 4 to 7.6 %). The rate errors were calculated by dividing the volume errors by the time interval between two acquisition dates. The rate errors do not exceed 0.7 m^3/sec.

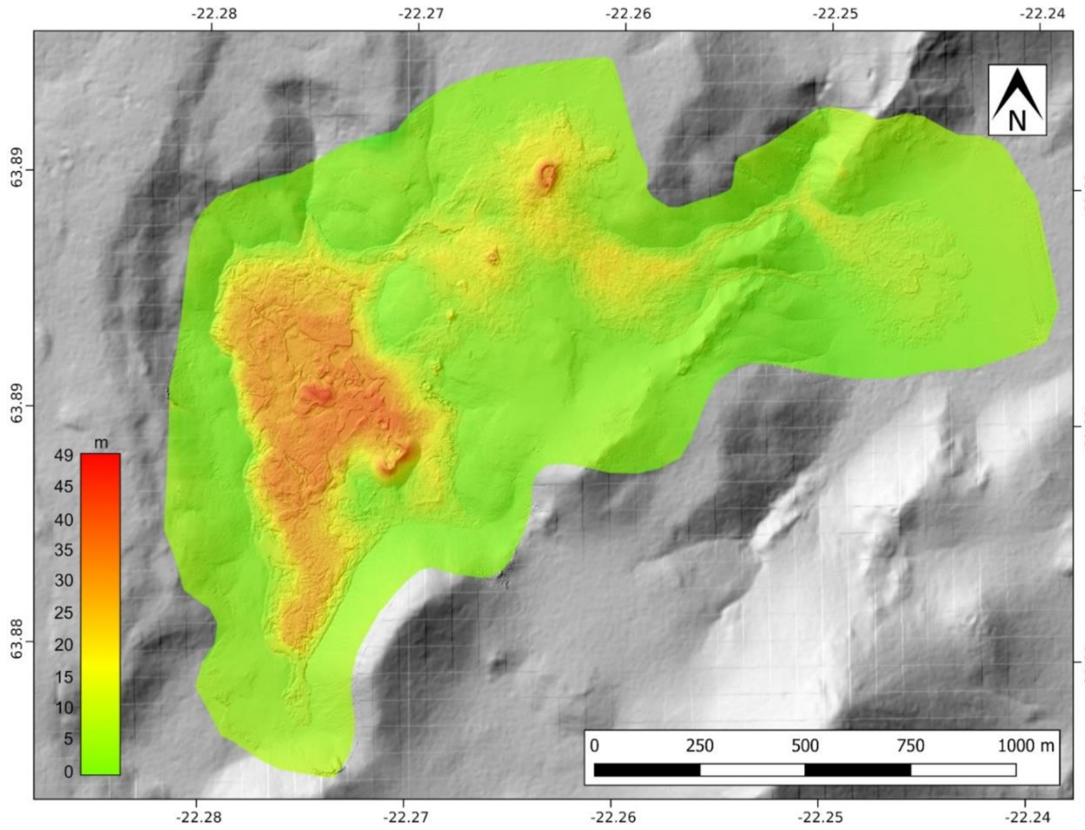

Fig. S3. Height difference map comparing the pre-eruption surface (before 19.03.2021) and surface derived from the 13.04.2021 UAV data showing the material distribution over the first stage of the eruption. Green indicates no major change, and yellow and red indicate material addition. The map is superimposed on the hillshade visualization of the DEM combined from the 2016 DEM created by the National Land Survey of Iceland LMI (http://atlas.lmi.is/mapview/?application=DEM) and from the 13.04.2021 drone DEM. The lava volume reached 13 million m^3 by 13.04.2021, and the discharge rate over the period was 6 m^3/sec.

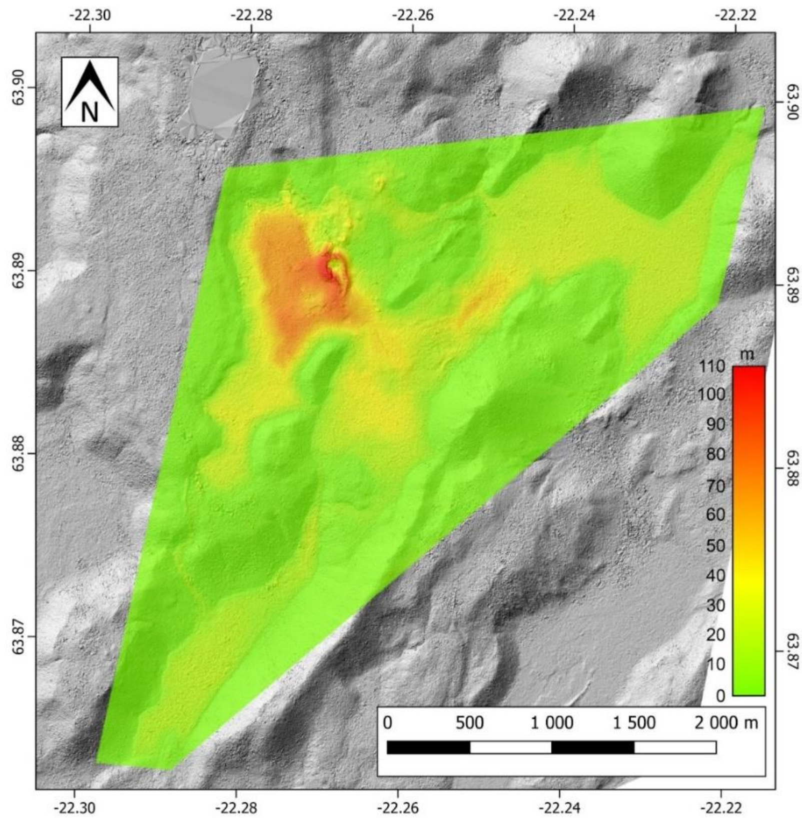

Fig. S4. Height difference map comparing the pre-eruption surface and surface derived from the 02.07.2021 Pleiades data. Green indicates no major change, and yellow and red indicate material addition. The map is superimposed on the hillshade visualization of the DEM built from the Pleiades data. The lava volume increase since 13.04.2021 was 68 million m^3 and the total lava volume reached 83 million m^3 by 02.07.2021. The discharge rate was 9.8 m^3/sec over the 13.04.2021-02.07.2021 period and 9.2 m^3/sec over the whole 19.03.2021-02.07.2021 period of the measurements.

Derived parameters:

<u>2016 base DEM (19.03.2021) – 13.04.2021 (UAV DEM)</u>

Area: 811,700 m^2
Volume: 12,858,000 m^3
Volume error: 974,000 m^3 (7.6%)
Rate: 514,300 m^3/day; 6 m^3/sec
Rate error: 39,000 m^3/day; 0.5 m^3/sec

<u>13.04.2021 (UAV DEM) – 02.07.2021 (Pleiades DEM)</u>

Area: 4,728,000 m^2
Volume: 68,063,000 m^3
Volume error: 4,823,000 m^3 (7.1 %)
Rate: 851,000 m^3/day; 9.8 m^3/sec
Rate error: 60,300 m^3/day; 0.7 m^3/sec

<u>2016 base DEM (19.03.2021) – 02.07.2021 (Pleiades DEM)</u>

Area: 4,728,000 m^2

Volume: 83,434,000 m^3

Volume error: 3,310,000 m^3 (4%)

Rate for the whole period of measurements (19.03.2021-02.07.2021): 794,600 m^3/day; 9.2 m^3/sec

Rate error: 7,600 m^3/day; 0.1 m^3/sec

Supplementary references

1. Sæmundsson, K., Sigurgeirsson, M. & Friðleifsson, G. Ó. Geology and structure of the Reykjanes volcanic system, Iceland. *J. Volcanol. Geotherm. Res.* **391**, 106501 (2020).
2. Shorttle, O. *et al.* Fe-XANES analyses of Reykjanes Ridge basalts: Implications for oceanic crust's role in the solid Earth oxygen cycle. *Earth Planet. Sci. Lett.* **427**, 272–285 (2015).

Supplementary Table 1: Sample information

Sample name	Latitude	Longitude	Eruption date	Sampling date	Notes	Days **
20210330-002	63.8876	-22.2665	20 March 2021	30 March 2021	Pahoehoe lava selvage.	1
IC-GEL-PAH-Vent1-01 (Fagra S1)*	63.8875	-22.2717	22 March 2021	27 March 2021	Pahoehoe lava.	3
20210320-002	63.8904	-22.2755	20 March 2021	20 March 2021	Ropey pahoehoe lava selvage.	1
20210321-003	63.8866	-22.2744	20 March 2021	21 March 2021	Lava selvage (tip of inactive lava flow).	1
20210325-003	63.8898	-22.2776	25 March 2021	25 March 2021	Active spiney pahoehoe lava flow.	6
IC-GEL-PAH-Vent1-02 (Fagra S2)*	63.8910	-22.2705	27 March 2021	27 March 2021	Interior of active lava flow.	8
20210327-002	63.8884	-22.2772	27 March 2021	27 March 2021	Interior of active spiney lava flow.	8
20210330-001	63.8874	-22.2764	30 March 2021	30 March 2021	Active spiney pahoehoe lava flow.	11
20210331-002	63.8871	-22.2724	31 March 2021	31 March 2021	Interior of active aa lava flow.	12
AH020421-02	63.8830	-22.2487	02 April 2021	02 April 2021	Tephra from vent 1b.	14
IC-GEL-PAH-Vent1-03 (Fagra 1)*	63.8897	-22.2777	02 April 2021	02 April 2021	Active pahoehoe lava flow.	14
IC-GEL-PAH-Vent1-04 (Fagra 2)*	63.8878	-22.2771	02 April 2021	02 April 2021	Active pahoehoe lava flow.	14
20210402-003	63.8861	-22.2760	02 April 2021	02 April 2021	Active pahoehoe lava flow.	14
05042021-010	63.89016	-22.26765	05 April 2021	05 April 2021	Tephra	17
20210406-004	63.8916	-22.2457	06 April 2021	06 April 2021	Interior of active pahoehoe lava lobe.	18
IC-GEL-PAH-Vent3-01 (Fagra 5)*	63.8923	-22.2689	08 April 2021	09 April 2021	Pahoehoe lava.	20
IC-GEL-PAH-Vent2-02 (Fagra 4)*	63.8951	-22.2660	09 April 2021	09 April 2021	Active pahoehoe lava flow.	21
IC-GEL-BLK-Vent1-05 (Fagra 6)*	63.8838	-22.2755	09 April 2021	09 April 2021	Active pahoehoe lava flow.	21
20210409-005	63.8927	-22.2500	09 April 2021	09 April 2021	Active rubbly pahoehoe lava flow.	21
IC-GEL-PAH-Vent4-01 (Fagra 7)*	63.8931	-22.2645	10 April 2021	10 April 2021	Active pahoehoe lava flow.	22
20210416-001 (1)	63.8867	-22.2631	16 April 2021	16 April 2021	Active rubbly pahoehoe lava flow.	28
20210416-001 (2)	63.8866	-22.2631	16 April 2021	16 April 2021	Active pahoehoe lava flow.	28
20210420-002	63.8873	-22.2680	20 April 2021	20 April 2021	Active pahoehoe lava flow from pond overflow.	32

Sample name	Latitude	Longitude	Eruption date	Sampling date	Notes	Days **
20210421-002	63.8833	-22.2604	21 April 2021	21 April 2021	Interior of active rubbly pahoehoe lava flow.	33
250421-01	63.8663	-21.1476	25 April 2021	25 April 2021	Lava block from rubbly pahoehoe/aa.	37
20210427-007	63.8831	-22.2676	27 April 2021	27 April 2021	Squeeze out of toothpaste pahoehoe from rubbly pahoehoe lava flow.	39
20210429-001	63.8841	-22.2584	29 April 2021	29 April 2021	Active spiney pahoehoe lava flow.	41
AH20210502-1	63.8925	-22.2430	01 May 2021	02 May 2021	Pahoehoe lava.	43
04052020-002	63.8865	-22.2772	04 May 2021	04 May 2021	Active rubbly pahoehoe lava flow.	46
20210506-004	63.8922	-22.2365	06 May 2021	06 May 2021	Active rubbly pahoehoe lava flow.	48
20210509-002	63.8808	-22.2585	09 May 2021	09 May 2021	Shelly pahoehoe lava.	51
20210511-002	63.8807	-22.2646	11 May 2021	11 May 2021	Active pahoehoe lava flow.	53
WM20210601-002	63.8907	-22.2309	01 June 2021	01 June 2021	Ropey pahoehoe lava flow	74
WM20210603-003	63.8907	-22.2309	03 June 2021	03 June 2021	Active pahoehoe lava flow.	76
WM20210607-001	63.8692	-22.2812	07 June 2021	07 June 2021	Active spiney pahoehoe lava flow.	80
WW20210608-001	63.8678	-22.2847	08 June 2021	08 June 2021	Active ropey pahoehoe lava flow.	81
JSP20210611-002	63.8872	-22.2305	11 June 2021	11 June 2021	Active ropey pahoehoe lava flow.	84
WM20210613-001	63.8692	-22.2854	13 June 2021	13 June 2021	Active aa lava flow.	86
WM20210614-001	63.8757	-22.2862	14 June 2021	14 June 2021	Active aa lava flow.	87
JSP20210618-001	63.8638	-22.2932	18 June 2021	18 June 2021	Active pahoehoe lava flow.	91
HKT20210620-001	63.8980	-22.2176	20 June 2021	20 June 2021	Active pahoehoe lava flow.	93
WM20210624-001	63.8881	-22.2272	24 June 2021	24 June 2021	Active ropey pahoehoe lava flow.	97
WM20210629-001	63.8635	-22.2939	29 June 2021	29 June 2021	Active pahoehoe lava flow.	102
WM20210717-002	63.89453	-22.22635	17 July 2021	17 July 2021	Active pahoehoe lava flow.	120
WM20210722-001	63.88877	-22.23634	22 July 2021	22 July 2021	Active pahoehoe lava flow.	125
WM20210803-002	63.89726	-22.2498	3 August 2021	3 August 2021	Pahoehoe lava.	137
JSP20210820-002	63.89457	-22.25814	20 August 2021	20 August 2021	Pahoehoe lava.	154

Sample name	Latitude	Longitude	Eruption date	Sampling date	Notes	Days **
WM20210826-001	63.86800	-22.28089	26 August 2021	26 August 2021	Active pahoehoe lava.	160

*Samples collected by GFZ; all others collected by the University of Iceland.
**Days since eruption start. March 19th = day zero.
